# Adolescent substance use in Costa Rica: findings from a national survey among secondary school students

**DOI:** 10.3389/fpubh.2025.1655355

**Published:** 2025-09-17

**Authors:** Pablo Montero-Zamora, Andrea Lopez-Soto, Jeancarlo Cordoba, Esmeralda Ramirez

**Affiliations:** ^1^Department of Kinesiology and Health Education, University of Texas at Austin, Austin, TX, United States; ^2^School of Public Health, University of Costa Rica, San José, Costa Rica

**Keywords:** adolescent health, substance use, Costa Rica, e-cigarettes, national surveys

## Abstract

**Background:**

Adolescent substance use (alcohol, tobacco, e-cigarette, marijuana, and other illegal drug use) is a growing public health concern in Latin America. In Central America, Costa Rica consistently reports among the greatest rates of alcohol and marijuana use among secondary school students in the region. However, nationally representative, peer-reviewed studies examining prevalence and etiologic factors remain scarce. This study aimed to examine the prevalence, distribution and associated etiological factors of substance use among Costa Rican adolescents.

**Methods:**

We analyzed data from the 2021 VI National Survey on Psychoactive Substance Use in the Secondary School Population, a cross-sectional, nationally representative survey of 3,524 students (weighted *N* ≈ 354,330) aged 11–20 years. We described the lifetime prevalence of alcohol, tobacco, e-cigarette, marijuana, and other illegal drug use and examined associations with sociodemographic, familial, and emotional distress variables using multivariate logistic regression.

**Results:**

Alcohol was the most commonly used substance (55.9%), followed by e-cigarettes (13.3%), tobacco (9.8%), other illegal drugs (7.4%), and marijuana (7.2%). Substance use was associated with older age, higher weekly allowance, working while studying, and family substance use. Emotional distress indicators—loneliness, sadness, and suicidal thoughts—were significantly associated with alcohol, tobacco, and other illegal drug use. Parental school monitoring was protective against alcohol use. Notably, e-cigarette use was inversely associated with suicidal thoughts and family smoking.

**Conclusion:**

This is the first peer-reviewed study to report nationally representative estimates of adolescent substance use in Costa Rica. Findings underscore the multifactorial etiology of substance use and highlight the need for culturally tailored, evidence-based prevention interventions in Costa Rica and Central America.

## 1 Introduction

In 2021, more than 480,000 deaths in Latin America and the Caribbean were attributed to substance use (i.e., alcohol, tobacco, marijuana, and other illegal drugs [i.e., amphetamines, cocaine, hallucinogens, inhalants, and opioids]), with approximately 30% occurring in Central Latin American countries—underscoring the scale of this public health problem in the region, according to the latest Global Burden of Disease study (1). In Costa Rica, alcohol, tobacco, and illegal drug use accounted for three of the fifteen leading behavioral risk factors contributing to the number of disability-adjusted life years (DALYs) lost in 2021, with tobacco and alcohol use ranking seventh and eighth, respectively (1). For instance, nearly 2,700 deaths in Costa Rica were attributed to substance use, with approximately 80% occurring among males (1). When focusing on the population aged 10–19 years, substance use has been the leading behavioral risk factor for mortality and disability in Costa Rica over the past two decades—a trend also observed across Central America—underscoring the urgent need for prevention research and intervention in the region (1).

Costa Rica has consistently ranked among the Central American countries with the highest prevalence of alcohol and marijuana use among secondary school students. Prevalence levels for these substances are typically comparable to, or slightly lower than, those of Panama and Belize and generally higher than those of Guatemala and Honduras, although not consistently higher than those of El Salvador. For instance, according to available national school-based surveys in Central America conducted between 2011 and 2018, past-year alcohol use among students was reported at 27.0% in Costa Rica, compared to 28.9% in Panama and 27.9% in Belize. In contrast, lower rates were observed in Guatemala (16.8%), Honduras (15.8%), and El Salvador (10.3%). Similarly, lifetime marijuana use was reported by 9.4% of students in Costa Rica, compared to 24.3% in Belize and 8.7% in Panama. While usage was lower in Guatemala (5.1%) and Honduras (2.9%), it was notably higher in El Salvador (15.1%) (2–7).

A growing body of research identifies a range of etiologic factors (i.e., risk and protective factors) associated with adolescent substance use. Early initiation, particularly before age 15, is among the most robust predictors of heavier alcohol and illegal drug use later in adolescence and adulthood (8–10). Substance use generally increases during the transition from middle to high school, with prevalence declining substantially after age 21 (8). Sex differences have also been documented, with women being less likely than men to use substances (11, 12). However, recent evidence suggests that this sex gap in substance use has narrowed (13). Further, several contextual and behavioral factors—including living in urban areas, working while attending school, and having access to discretionary spending money—have been associated with increased substance use risk (14, 15). Social environments may also play a key role, with the number of friends reported by an adolescent (16), substance use in the family (17), and parental involvement (18) emerging as relevant etiologic factors. Additionally, internalizing symptoms related to emotional distress—such as loneliness (19) and sadness (20), as well as suicidal thoughts (21)—have been associated with increased vulnerability to substance use during adolescence. These etiologic factors inform the selection of variables examined in the present study.

To effectively reduce substance use and its related harms among adolescents in Costa Rica—and across Central America—it is essential to design, implement, and evaluate prevention interventions informed by an understanding of the etiologic factors that may influence these behaviors. However, as of the time of this study, epidemiological and etiological research on substance use among secondary school students remains limited in the region and is particularly scarce in Costa Rica. One of the few available studies using a nationally representative sample of Costa Rican youth (15–24 years) identified factors such as age, sex, geographic region, discrimination, sexual victimization, and community involvement as being associated with substance use (22). Expanding on this, this study utilized data collected prior to the COVID-19 pandemic and focused on out-of-school adolescents, which limits its applicability to the school-based population. To date, no specific analyses have been conducted on Costa Rican adolescents who are enrolled in school using representative national datasets. While government agencies have released descriptive reports based on national survey data collected in high schools (2, 23), there is still a lack of rigorous, peer-reviewed, population-based studies examining the prevalence and etiologic factors associated with substance use among Costa Rican students. Such research is crucial for informing the development of effective policies and evidence-based interventions tailored to this population.

Therefore, this study used the most recent student data available in Costa Rica—the VI National Survey on Psychoactive Substance Use in the Secondary School Population (VI-NSPSSU) (23)—to (a) describe the national prevalence of alcohol, tobacco, e-cigarette, marijuana, and other illegal drug use among secondary school students in Costa Rica; (b) examine the geographic and sociodemographic distribution of substance use across regions and population subgroups; and (c) explore the association between substance use and potential etiologic factors, including sociodemographic characteristics (e.g., age, sex), number of friends, substance use in the family, family involvement (e.g., parental rule-setting, parental affection, and parental school monitoring), and emotional distress (e.g., loneliness, sadness, suicidal thoughts).

## 2 Methods

### 2.1 Participants

Study participants included students enrolled in Costa Rica’s daytime secondary education system during 2021 (i.e., students attending schools operating during standard morning and afternoon hours). The sampling frame was based on official enrollment data from the Costa Rican Ministry of Public Education. Students enrolled in night schools were not included, as were schools that did not offer continuous enrollment across grades 7th through 11th as of 2020. The final analytic sample consisted of 3,524 students, corresponding to a nationally representative sample of more than 300,000 adolescents enrolled in the formal education system after applying sampling weights. Sample descriptive characteristics–including frequency distributions–are presented in Table 1.

**Table 1:** Descriptive statistics of predictors for substance use among secondary students in Costa Rica, 2021 (*N* = 354,330).

Variables	Population	
	%	Mean (*SD*)
Total	100.0	
Sex		
Men	45.6	
Women	54.4	
Age (Years)	–	15.2 (1.66)
Province		
San Jose	27.8	
Alajuela	18.7	
Heredia	10.3	
Cartago	10.8	
Guanacaste	9.7	
Puntarenas	12.6	
Limon	10.2	
Rural high school	32.4	
Weekly allowance (Dollars)		
None	39.7	
≤ 20 dollars	46.7	
> 20 dollars	13.6	
Work while studying	11.3	
Number of friends	–	1.98 (1.07)
Substance use in the family		
Family binge drinking	10.8	
Family smoking	16.6	
Family illegal drug use	7.7	
Family involvement		
Parental rule-setting	76.5	
Parental affection	85.5	
Parental school monitoring	84.4	
Emotional distress		
Loneliness	64.0	
Sadness or hopeless	39.3	
Suicidal thoughts	16.1	

% = Prevalence in valid cases. *SD* = Standard deviation. Ref = reference.

### 2.2 Study design and survey

This cross-sectional study corresponds to a secondary data analysis of the VI-NSPSSU (23). The survey was administered by the Costa Rican Institute on Alcoholism and Drug Dependence (IAFA, representing its initials in Spanish), a national governmental agency responsible for leading the country’s surveillance of student substance use. The VI-NSPSSU is part of an ongoing effort, dating back to 2006, to monitor adolescent substance use and inform youth-focused policies and programs. The VI-NSPSSU was a nationally representative survey designed to assess the prevalence of alcohol, tobacco, e-cigarette, marijuana, and other illegal drug use (i.e., cocaine, crack, inhalants, mushrooms, and ketamine) among adolescents, as well as relevant individual-, school-, family-, and community-level- indicators related to substance use (e.g., demographic characteristics, family substance use, emotional distress, and perceived family involvement).

### 2.3 Sampling procedures

The VI-NSPSSU sampling process consisted in a two-stage stratified area probability sampling design (24) involving 3,524 students (i.e., elements) drawn from 60 schools (i.e., secondary sampling units or clusters) across 10 primary sampling units (i.e., geostatistical IAFA-defined regions [i.e., Brunca, Central East, Central North Alajuela, Central North Heredia, Central West, Central Southeast, Central Southwest, Chorotega, Huetar Caribbean, and Central Pacific]). In the first stage, all daytime secondary schools were stratified by 10 IAFA-defined administrative regions and further divided by school grade (i.e., 7th-11th), resulting in 20 strata. Schools were selected without replacement with probability proportional to size (25). In the second stage, one classroom section was randomly selected from each grade level within each participating school, and all students present were invited to participate. The final weighted sample for analysis corresponded to 354,330 students.

### 2.4 Procedures

The VI-NSPSSU data were collected via an online survey during school hours using a platform (i.e., Typeform) designed for administering surveys in the participating schools, with teachers granting access and supervising completion. The survey took approximately 55 min to complete. Each participating school received a copy of the survey along with access to the corresponding data repository for their records. The questionnaire was accessible via computer, tablet, or smartphone. Students who provided assent completed the survey online. Paper-based survey administration was offered when internet connectivity was limited (< 2% of schools). Access to the de-identified dataset from the VI-NSPSSU was granted by the IAFA on February 4, 2025, following a formal request submitted by the corresponding author. Additionally, all research procedures received approval from an Institutional Review Board at the corresponding author’s affiliated institution prior to the start of the research.

### 2.5 Measures

#### 2.5.1 Substance use

We assessed lifetime use of alcohol, tobacco (i.e., cigarettes, cigars, pipes, hand-rolled cigars, or hookah), e-cigarettes, marijuana, and other illegal drugs (i.e., cocaine, crack, ketamine, acids, ecstasy, methamphetamines, heroin, inhalants, and hallucinogenic plants) using four independent items. Sample items included for (a) alcohol—“Which of these alcoholic beverages have you ever had in your life (e.g., beer, wine, rum)?”; and (b) tobacco—“Have you ever smoked or tried smoking cigarettes, even just one or two puffs?” and “Have you ever tried other tobacco products besides cigarettes (e.g., cigars, pipes, hand-rolled cigars, or hookah)?” Responses were dichotomized (*yes* = 1, *no* = 0). Marijuana was analyzed separately to reflect recent policy changes and changing society’s perspective on marijuana (26). E-cigarettes were also analyzed separately, given their increasing prevalence worldwide (27).

#### 2.5.2 Sociodemographic

Participants self-reported sociodemographic characteristics, including sex (*male* = 0, *female* = 1), age (*M* = 15.2, *SD* = 1.66), and weekly allowance (*none* = 0, ≤ $20 = 1, > $20 = 2). They were also asked whether they engaged in paid work while attending school—such as hourly or occasional employment— which was coded dichotomously (*yes* = 1, *no* = 0). Additional variables included (a) the location of the province of each participant’s high school (*San Jose* = 0, *Alajuela* = 1, *Heredia* = 2, *Cartago* = 3, *Guanacaste* = 4, *Puntarenas* = 5, and *Limon* = 6); and (b) whether the school was located in a rural area (*yes* = 1, *no* = 0). Rural status was determined based on the district where each school was situated, using the urbanization classification provided by the Costa Rican National Institute of Statistics and Censuses (28).

#### 2.5.3 Number of friends

Participants reported the number of close friends using a single item asking, “In total, how many very close friends do you currently have?” This single-item measure served as a proxy for the size of adolescents’ close friendship networks.

#### 2.5.4 Substance use in the family

Exposure to substance use in the family was measured using three dichotomous single items for (a) family binge drinking—“Does anyone in your household drink excessive alcohol or get drunk frequently?”; (b) family smoking—“Does anyone in your household smoke tobacco or use nicotine?”; and (c) family illegal drug use —“Does anyone in your household use any of the following substances: inhalants, marijuana, cocaine, crack, hallucinogens, or ecstasy?” Responses to each item were included as dummy variables in the analyses (*yes* = 1, *no* = 0).

#### 2.5.5 Perceived family involvement

Perceived family involvement was assessed using three single items, each capturing a specific aspect of parental involvement (a) parental rule-setting—“Do either of your parents set a time for you to be home?; (b) parental affection— “Do either of your parents make you feel loved and cared for?”; and (c) parental school monitoring—“Do either of your parents monitor your school activities (e.g., homework, grades, meetings with teachers)?” Responses were coded dichotomously (*yes* = 1, *no* = 0).

#### 2.5.6 Emotional distress

Emotional distress was assessed using three single items, each reflecting a separate aspect of distress (a) loneliness—“In the past year, how often have you felt lonely?”; (b) feelings of sadness and hopelessness—“In the past year, were there two consecutive weeks when you stopped doing your usual activities because you felt very sad or hopeless?”; and (c) suicidal thoughts—“In the past year, have you seriously considered attempting suicide?” Responses to each item were coded dichotomously (*yes* = 1, *no* = 0).

### 2.6 Data analysis

We used Stata 17 (29) to address the VI-NSPSSU sampling design (i.e., two-stage stratified area probability sampling), which stratified the sampled high school students and the high schools into the 10 geostatistical regions (i.e., primary sampling units). First, we conducted descriptive analyses to estimate the population-level prevalence of substance use across various sociodemographic subgroups. Next, we conducted a series of multivariate survey-weighed logistic regression analyses (30) to examine associations between our dependent variables (i.e., the prevalence of lifetime use of alcohol, tobacco, e-cigarettes, marijuana, and other illegal drugs) and independent variables (i.e., sociodemographic characteristics, number of friends, substance use in the family, perceived family involvement, and emotional distress). All final models were interpreted using adjusted odds ratios (AOR). Missing values showed no clear pattern of systematic non-response, suggesting data were missing at random (31). Survey-weighted logistic regression analyses were conducted using listwise deletion (i.e., only complete cases), which, under the assumption of random missingness, are expected to yield unbiased estimates.

## 3 Results

### 3.1 Substance use prevalence

Table 2 presents lifetime substance use prevalence among Costa Rican secondary school students, disaggregated by sociodemographic characteristics. Overall, alcohol was the most commonly used substance, followed by e-cigarettes, tobacco, other illegal drugs, and marijuana. Female students reported greater prevalence of alcohol and cigarette use compared to males, whereas males showed greater use of e-cigarettes, marijuana, and other illegal drugs. Substance use generally increased with age for all substances except e-cigarettes, which exhibited an inverted U-shaped pattern, with lower prevalence observed in the oldest age group (18–20 years).

**Table 2:** Substance use populational prevalence across sociodemographic subgroups among secondary students in Costa Rica, 2021.

Variables	Prevalence (%)				
	Alcohol	Tobacco	E-cigarettes	Marijuana	Other illegal drugs
Total	55.9	9.8	13.3	7.2	7.4
Sex					
Men	53.5	9.6	14.9	8.8	9.0
Women	61.6	9.9	12.2	5.7	6.5
Age					
11–14	39.4	3.0	13.3	1.8	7.6
15–17	67.1	12.6	14.4	9.9	7.4
18–20	82.6	22.2	8.2	14.3	9.3
Province					
San Jose	63.1	11.8	11.9	8.5	8.6
Alajuela	55.4	8.8	10.5	4.6	5.5
Heredia	51.4	8.7	14.3	2.7	5.0
Cartago	57.6	10.9	15.7	13.1	9.3
Guanacaste	49.4	7.2	21.5	4.8	6.4
Puntarenas	54.9	11.9	14.9	7.6	9.1
Limon	47.1	5.9	8.3	5.1	6.8
Rural high-school					
No	57.3	9.6	15.0	7.6	7.9
Yes	53.0	10.3	9.6	6.1	6.3
Weekly allowance (Dollars)					
None	53.1	7.8	13.5	5.5	6.9
≤ 20 dollars	56.8	10.1	13.5	6.8	7.5
> 20 dollars	72.0	17.0	14.1	16.0	14.8
Work while studying					
No	55.8	9.2	13.6	6.7	7.0
Yes	75.0	14.6	12.1	10.9	12.6

Regarding geographical location, alcohol was the most prevalent substance among students across all seven provinces. San Jose reported the greatest alcohol use prevalence, while tobacco use was most common in Puntarenas. Additionally, e-cigarette use was highest in Guanacaste, and marijuana and other illegal drug use were most prevalent in Cartago. In contrast, Limon consistently showed the lowest prevalence of alcohol, tobacco, and e-cigarettes. Further, Heredia reported less use of marijuana and other illegal drugs. Similarly, substance use–except for tobacco–was lower among students attending rural high schools than among those attending high schools in urban areas. Students with weekly allowances over $20 and those working while studying reported greater substance use across most substances, except e-cigarette use, which was lower among working students. See Table 2 for details.

### 3.2 Logistic regression findings

#### 3.2.1 Alcohol

As presented in Table 3, alcohol use among secondary school students was significantly associated with multiple etiologic factors (all *p* values < 0.05). Specifically, women were more likely to report lifetime alcohol use compared to men (AOR = 1.26, 95% CI = 1.03–1.55) and age was positively associated with alcohol use (AOR = 1.51, 95% CI = 1.39–1.65). Students in Heredia had significantly lower odds of alcohol use compared to those in San Jose (AOR = 0.54, 95% CI = 0.30–0.98). No other geographical differences were observed. In terms of economic factors, students who received more than $20 in weekly allowance had nearly twice the odds of alcohol use as those with no allowance (AOR = 1.95, 95% CI = 1.31–2.89). Similarly, students engaged in paid work were more than twice as likely to report alcohol use compared to those who did not work (AOR = 2.25, 95% CI = 1.46–3.46).

**Table 3:** Parameters estimates and confidence intervals for predictors of substance use prevalence among secondary students in Costa Rica, 2021.

Variables	Alcohol		Tobacco		E-Cigarettes		Marijuana		Other illegal drugs	
	AOR	95%CI	AOR	95%CI	AOR	95%CI	AOR	95%CI	AOR	95%CI
Sex (Ref = Men)										
Women	**1.26** ^*^	1.03–1.55	0.96	0.62–1.47	0.90	0.68–1.19	**0.58** ^*^	0.37–0.91	**0.55** ^*^	0.34–0.91
Age	**1.51** ^***^	1.39–1.65	**1.59** ^***^	1.43–1.78	0.94	0.83–1.05	**1.60** ^***^	1.43–1.78	1.01	0.91–1.13
Province (Ref = San Jose)										
Alajuela	0.82	0.37–1.83	0.79	0.34–1.83	0.99	0.55–1.77	**0.61** ^*^	0.38–0.98	0.73	0.36–1.45
Heredia	**0.54** ^*^	0.30–0.98	**0.66** ^*^	0.46–0.93	1.22	0.69–2.15	**0.32** ^**^	0.15–0.66	0.61	0.32–1.17
Cartago	1.01	0.52–1.94	0.84	0.50–1.39	1.47	0.54–4.00	2.02	0.99–4.13	1.14	0.51–2.56
Guanacaste	0.67	0.33–1.36	0.54	0.28–1.04	0.97	1.16–3.34	**0.52** ^*^	0.28–0.94	0.76	0.40–1.44
Puntarenas	0.89	0.51–1.57	0.75	0.44–1.27	1.55	0.88–2.74	0.93	0.56–1.54	1.02	0.51–2.03
Limon	0.74	0.40–1.38	**0.54** ^*^	0.32–0.90	0.51	0.23–1.13	0.80	0.34–1.90	1.01	0.48–2.12
Rural high school (No = 0; Yes = 1; Ref = No)	1.07	0.73–1.56	1.30	0.87–1.94	**0.62** ^*^	0.41–0.93	0.79	0.51–1.23	0.93	0.65–1.34
Weekly allowance (Dollars; Ref = None)										
≤ 20 dollars	1.06	0.82–1.37	1.36	0.81–2.28	1.00	0.70–1.44	1.19	0.77–1.83	1.11	0.61–2.04
> 20 dollars	**1.95** ^**^	1.31–2.89	**2.09** ^*^	1.06–4.10	0.97	0.62–1.54	**2.56** ^**^	1.41–4.66	1.98	0.96–4.06
Work while studying (No = 0; Yes = 1; Ref = No)	**2.25** ^***^	1.46–3.46	1.31	0.77–2.22	1.12	0.61–2.03	1.12	0.59–2.11	1.46	0.73–2.90
Number of friends	**1.21** ^***^	1.09–1.35	0.94	0.79–1.11	1.13	1.00–1.28	0.98	0.82–1.17	1.06	0.84–1.33
Substance use in the family (No = 0; Yes = 1; Ref = No)										
Family binge drinking	**2.03** ^***^	1.35–3.06	1.23	0.76–2.01	1.01	0.58–1.78	0.66	0.36–1.21	1.92	0.91–4.05
Family smoking	1.29	0.96–1.73	1.34	0.87–2.08	**0.66** ^*^	0.45–0.97	**1.56** ^*^	1.03–2.37	0.94	0.49–1.77
Family illegal drug use	**2.93** ^**^	1.42–6.07	**3.12** ^***^	1.83–5.32	1.47	0.70–3.05	**4.87** ^***^	2.30–10.31	**2.87** ^***^	1.73–4.77
Family involvement (No = 0; Yes = 1; Ref = No)										
Parental rule-setting	1.24	0.89–1.73	0.79	0.49–1.29	0.79	0.52–1.19	0.72	0.43–1.19	0.79	0.43–1.43
Parental affection	1.00	0.71–1.41	1.06	0.70–1.60	0.82	0.49–1.39	1.04	0.59–1.86	0.75	0.40–1.40
Parental school monitoring	**0.63** ^*^	0.42–0.96	0.74	0.42–1.31	1.34	0.59–3.08	0.98	0.46–2.12	1.08	0.59–1.96
Emotional distress (No = 0; Yes = 1; Ref = No)										
Loneliness	**1.43** ^*^	1.02–2.01	**2.56** ^**^	1.45–4.50	1.08	0.65–1.79	1.38	0.85–2.26	1.09	0.68–1.75
Sadness or hopeless	**1.55** ^*^	1.05–2.27	1.17	0.75–1.84	1.15	0.74–1.77	1.67	0.89–3.13	**2.68** ^***^	1.72–4.18
Suicidal thoughts	**1.87** ^***^	1.34–2.61	**1.61** ^*^	1.01–2.55	0.55*	0.33–0.91	1.46	0.81–2.65	**2.02** ^*^	1.16–3.53

AOR, Adjusted Odds Ratio. 95% CI, 95% Confidence Interval. Ref, reference.

*p < 0.05. ** *p* ≤ 0.01. *** *p* ≤ 0.001. Bolded values indicate statistically significant associations.

Alcohol use was positively associated with students’ reports of having more close friends (AOR = 1.21, 95% CI = 1.09–1.35). Further, alcohol use was associated with family binge drinking (AOR = 2.03, 95% CI = 1.35–3.06) and family use of illegal drugs (AOR = 2.93, 95% CI = 1.91–4.51). In contrast, parental school monitoring was associated with a reduced likelihood of alcohol use (AOR = 0.63, 95% CI = 0.42–0.96). Additionally, all three indicators of emotional distress were significantly associated with increased alcohol use (loneliness [AOR = 1.43, 95% CI = 1.02–2.01], sadness or hopelessness [AOR = 1.55, 95% CI = 1.05–2.27], and suicidal thoughts [AOR = 1.87, 95% CI = 1.34–2.61]).

#### 3.2.2 Tobacco and e-cigarettes

As shown in Table 3, tobacco use was positively associated with age (AOR = 1.59, 95% CI = 1.43–1.78), weekly allowance (AOR = 2.09, 95% CI = 1.06–4.10), family illegal drug use (AOR = 3.12, 95% CI = 1.83–5.32), loneliness (AOR = 2.56, 95% CI = 1.45–4.50), and suicidal thoughts (AOR = 1.61, 95% CI = 1.01–2.55). Compared to students in San Jose, those in Heredia (AOR = 0.66, 95% CI = 0.46–0.93) and Limon (AOR = 0.54, 95% CI = 0.32–0.90) had lower odds of tobacco use. In contrast, fewer factors were associated with e-cigarette use. Students attending rural schools had lower odds of e-cigarette use than their urban counterparts (AOR = 0.62, 95% CI = 0.41–0.93). Notably, e-cigarette use was less likely among students with family members who smoke (AOR = 0.66, 95% CI = 0.45–0.96) and those reporting suicidal thoughts (AOR = 0.55, 95% CI = 0.33–0.91)—both associations in the opposite direction of those observed for tobacco.

#### 3.2.3 Marijuana

Marijuana use among Costa Rican students was positively associated with age (AOR = 1.60, 95% CI = 1.43–1.78), weekly allowance over $20 (AOR = 2.56, 95% CI = 1.41–4.66), household smoking (AOR = 1.56, 95% CI = 1.03–2.37), and other illegal drug use in the family (AOR = 4.87, 95% CI = 2.30–10.31). Female students had significantly lower odds of marijuana use compared to male students (AOR = 0.58, 95% CI = 0.37–0.91). Compared to students in San Jose, those in Alajuela (AOR = 0.61, 95% CI = 0.38–0.98), Heredia (AOR = 0.32, 95% CI = 0.15–0.66), and Guanacaste (AOR = 0.52, 95% CI = 0.28–0.94) had lower chances of marijuana use.

#### 3.2.4 Other illegal drugs

The use of other illegal drugs was associated with several etiologic factors. Female students were less likely to use other illegal drugs compared to males (AOR = 0.55, 95% CI = 0.34–0.91). Students reporting illegal drug use within their families–including marijuana–were nearly three times more likely to use other illegal drugs themselves (AOR = 2.87, 95% CI = 1.73–4.77). Emotional distress was also associated with other illegal drug use since students who reported feeling sadness or hopeless in the past year were more likely to use other illegal drugs (AOR = 2.68, 95% CI = 1.72–4.18) and those who seriously considered attempting suicide in the past year were more likely to use other illegal drugs (AOR = 2.02, 95% CI = 1.16–3.53).

## 4 Discussion

To our knowledge, this is the first study to report national estimates of lifetime substance use and potential etiologic factors among secondary school students in Costa Rica. Alcohol was the most commonly reported substance, followed by e-cigarettes, then tobacco, other illegal drugs, and marijuana. These patterns—excluding e-cigarette use, which was not previously measured—are consistent with findings from Costa Rica’s 2018 National Student Survey, which also identified alcohol as the most frequently used substance (2). However, overall substance use prevalence declined between 2018 and 2021, with the largest reductions observed for alcohol (25%) and marijuana (23%). These decreases should be interpreted in light of the COVID-19 pandemic, as data for the present study were collected in 2021, a period of national restrictions that likely limited adolescents’ opportunities for substance use (32). For example, in the United States, nationally representative data also documented declines in adolescent substance use between 2019 and 2021, with alcohol use dropping by about 22% and marijuana use by 27%. These trends lend support to the idea that pandemic-related disruptions, such as social distancing measures, may have temporarily reduced adolescents’ access to substances (33). These trends support the hypothesis that pandemic-related disruptions, such as social distancing, temporarily reduced adolescents’ access to substances. As such, our findings may reflect substance use patterns specific to 2021 and may underestimate typical prevalence and trends in Costa Rica, limiting their generalizability to subsequent years.

Notably, this study is the first to analyze national data on e-cigarette use among students in Costa Rica. Although e-cigarette use was the second most prevalent substance among students in Costa Rica, its prevalence was lower—potentially due to pandemic-related effects—than reported in other countries in the Americas, such as Colombia (32%) (34), the United States (17%) (35), and Argentina (14.4%) (36). Nevertheless, the findings regarding e-cigarette use point to an emerging public health concern for Costa Rica and the broader Latin American region.

Concerning sociodemographic factors, men were more likely than women to report lifetime use of marijuana and other illegal drugs, whereas women were more likely to report lifetime alcohol use. This pattern aligns with growing evidence of a narrowing gender gap in adolescent substance use, with young women increasingly exhibiting rates comparable to—or exceeding—those of young men. For example, the 2021–2022 international report of the Health Behavior in School-aged Children highlights a significant shift in substance use patterns across 44 countries and regions in Europe, Central Asia, and Canada, challenging historical sex-related differences in consumption that have traditionally shown greater prevalence among men. The report indicates that by age 15, young women often catch up to young men in cigarette smoking and e-cigarette use and surpass them in alcohol consumption (13).

In addition, age was a consistent predictor across substances, with older adolescents generally reporting greater use—except for e-cigarettes and other illegal drugs—consistent with a previous study conducted in Costa Rica using a nationally representative youth sample (22). Finally, living in provinces outside the capital San Jose was associated with a lower likelihood of alcohol, tobacco, e-cigarette, and marijuana use—but not other illegal drug use. Similarly, studying in urban schools was associated with a lower prevalence of e-cigarette use. These findings likely reflect an association between greater population density and increased substance use in urban settings, consistent with prior research documenting higher rates of adolescent substance use in more populated areas (15). Findings may also be influenced by differences in substance availability. For instance, legal substances such as tobacco, might be more easily accessible in urban areas, potentially contributing to the observed patterns, while limited availability in rural settings may shape distinct substance use behaviors. Future studies should explore how ease of access to legal substances vis-à-vis illegal substances might influence patterns of consumption in Costa Rica.

Economic factors also influenced adolescent substance use, with Costa Rican students receiving a greater weekly allowance (i.e., > 20$ per week) showing an increased likelihood of alcohol, tobacco, and marijuana use. This finding is consistent with prior research from various countries demonstrating positive associations between disposable income and substance use behaviors. For example, studies of European school samples have found that greater weekly allowances are associated with increased use of both licit (i.e., alcohol and tobacco) and illicit (i.e., marijuana and ecstasy) substances (14, 37–39).

Further, working while studying was associated specifically with alcohol use, which aligns with prior research showing that part-time employment during adolescence is associated with increased alcohol consumption. For instance, Finnish adolescents working over 10 h per week had a significantly higher odds of reporting heavy drinking (40). Similarly, Canadian high school students with part-time jobs reported greater alcohol use (41). Income from part-time work has also been associated with licit substance use, such as alcohol and tobacco, possibly reflecting the structured nature of employment or personal traits like financial responsibility (38). These findings have implications for school- and family-level prevention. Including financial management education and opportunities for positive involvement—such as those offered in evidence-based parenting programs like *Guiding Good Choices* (42)—may be valuable components in adolescent prevention curricula in Costa Rica.

Findings confirmed that social environments significantly shape substance use patterns in Costa Rica. For instance, we found that students with more friends were more likely to have ever consumed alcohol. Prior research shows that larger adolescent social networks contribute to underage drinking (16). Although we did not directly ask about peer substance use, students reporting more friends probably have more close peers who drink, which is a well-established factor influencing youth alcohol use (43). Regarding substance use within the family environment, family binge drinking was associated only with alcohol use, consistent with a study of over 4,700 Finnish adolescents showing that parents’ drinking predicted their children’s alcohol use and intoxication (44). Additionally, family use of illegal drugs was associated with students’ use of all substances except e-cigarettes, a result supported by a 2022 meta-analysis reporting that both maternal and paternal substance use increase the likelihood of child drug use (45).

Surprisingly, students who had smokers in the family reported lower e-cigarette use, a finding that contradicts meta-analytic evidence suggesting a positive association between adolescent e-cigarette use and family smoking (46). Further research is needed to better understand the connection between social environments—particularly the family—and e-cigarette use among Costa Rican students. Finally, consistent with findings from a 12-year longitudinal study conducted in Iceland, which showed that parental monitoring significantly reduced alcohol use and intoxication among adolescents, our results also identified parental monitoring as an important protective factor in the family domain (47). In contrast, rule-setting and expressions of affection did not show a protective effect. Their high prevalence among parents (>80%) may have reduced variability and limited our ability to detect potential associations due to ceiling effects. Additionally, our ability to fully assess these family-level protective factors was constrained by the use of dichotomous indicators (i.e., single-items), which may have reduced the granularity of the information included in the analysis. Future studies would benefit from incorporating validated scales–developed with multiple tested items–to more accurately capture the nuances of these constructs.

Students’ emotional distress emerged as an important individual-level etiologic factor for alcohol use. Specifically, our findings suggest that the three indicators—loneliness, feelings of sadness or hopelessness, and suicidal thoughts—were each associated with more than a 40% increase in underage drinking among students in Costa Rica. Similar findings have been reported in a nationally representative school-based study in South Africa, where feelings of sadness or hopelessness were significantly associated with binge drinking among adolescents (20). Furthermore, our findings mirror other studies reporting that alcohol use was associated with suicidal ideation and attempts among Chinese students (48). Similar associations—particularly between suicidal thoughts and alcohol use—have also been observed in a study conducted in the United States among high school students (21).

When examining the association between emotional distress and tobacco use, results indicate greater tobacco use among students reporting increased loneliness and suicidal ideation. Similar studies using data drawn from the Global School-Based Student Health Survey–led by the World Health Organization–found the same positive associations in the context of loneliness vis-à-vis tobacco use among students in South Asia (49) and the Caribbean (i.e., Dominican Republic, Jamaica, Suriname, Trinidad and Tobago) (50). Furthermore, Dasagi et al. (51), using the nationally representative Youth Risk Behavior Surveillance Survey in the United States, found that heavy cigarette smoking increased suicide risk among adolescents.

In addition, our findings indicate that the use of other illegal drugs was associated with elevated reports of sadness or hopelessness and suicidal thoughts. To our knowledge, no studies have directly examined the association between other illegal drugs use and self-reported single-item measures of feelings such as sadness or hopelessness. However, akin associations have been reported in studies employing validated measures of depressive symptomatology—such as the Short Mood and Feelings Questionnaire (SMFQ) (52), which includes items related to these emotional states. For instance, a longitudinal study conducted in Australia using the SMFQ found that (a) early adolescent depressive symptoms predicted the frequency of mid-adolescent illicit drug use, and (b) mid-adolescent depressive symptoms predicted the frequency of illicit drug use in late adolescence (53). Importantly, a previous study among 2,284 Costa Rican students from 64 middle and high schools also found an association between suicidal thoughts and a measure of substance use that included illicit drugs, consistent with our findings.

Unexpectedly, e-cigarette use was less prevalent among students reporting suicidal thoughts—a finding that contrasts with meta-analytic evidence using studies from the United States, Canada, and South Korea, where adolescent e-cigarette use has been significantly and positively associated with suicidal ideation, planning, and attempts (54). As noted in this meta-analysis, cultural and social factors may shape the association between e-cigarette use and suicidal behaviors. For example, differences in attitudes toward mental health and e-cigarettes—variables not measured in this study and potentially important covariates—could influence both adolescents’ reporting of mental health issues and their engagement with these products, potentially explaining discrepancies across populations (54). Additionally, common sociodemographic predictors of substance use, such as age and sex, were unrelated to e-cigarette use, a pattern similarly observed in recent studies from Mexico (55). Overall, these inconsistencies underscore the need for further longitudinal research on the etiological and contextual determinants of adolescent e-cigarette use in Costa Rica.

### 4.1 Limitations and future directions

Our findings must be interpreted in light of several limitations. First, the cross-sectional nature of our design limits the ability to infer causality, as observed associations may be subject to temporal bias and unmeasured confounding. Longitudinal research that integrates individual-, family-, and community-level risk and protective factors is critically needed to elucidate causal pathways and to inform the development of contextually appropriate prevention initiatives in the country and the region. A second limitation is that data were collected during the COVID-19 pandemic, and as discussed earlier, substance use prevalence may not accurately reflect post-pandemic patterns. We emphasize the importance of sustaining nationally representative survey efforts in Costa Rica to provide more recent data, which will support future studies and analyses, thereby improving the surveillance and monitoring of substance use trends among Costa Rican students.

Third, the use of self-report measures can be susceptible to social desirability bias, with students potentially misrepresenting their substance use, family dynamics, and socioemotional status. Future research should corroborate self-reports with other sources (e.g., peers, teachers, or parents) and biomarkers (e.g., drug testing) to enhance the validity of findings. Finally, consistent with the previous limitation, we emphasize the importance of using validated, standardized measures based on multiple items—rather than single items of institutional interest—to assess multilevel risk (e.g., emotional distress) and protective (e.g., perceived family involvement) factors. We strongly recommend that future studies adopt multi-item, validated scales to improve the reliability and validity of assessments, enhance decision-making and surveillance, and provide a more accurate understanding of the etiology of substance use in Costa Rica.

## 5 Conclusion

Despite these limitations, this study is the first to examine nationally representative estimates of lifetime substance use and explore associated etiologic factors, specifically among secondary school students in Costa Rica, using data from the VI-NSPSSU. Our results broadly support hypotheses derived from research conducted in other countries, indicating that substance use patterns in Costa Rica share many similarities with those observed in developed countries in North America and Europe. Notably, alcohol remains the most commonly used substance, followed by emerging e-cigarette use, with tobacco, other illicit drugs, and marijuana reported less frequently. Sociodemographic factors such as age, sex, geographic location, and economic indicators significantly influenced substance use prevalence, consistent with international findings. The influence of social environments—including peer networks and family substance use—and individual factors such as emotional distress further underscore the multifaceted nature of adolescent substance use etiology in Costa Rica.

Key exceptions to general patterns emerged, particularly in the associations involving e-cigarette use, family smoking, and suicidal thoughts, suggesting culturally and contextually specific dynamics that warrant further research. By documenting both established and country-specific potential etiologic factors, this study provides critical evidence to guide the development, implementation, and evaluation of culturally tailored, evidence-based prevention interventions that effectively reduce adolescent substance use and its related harms in Costa Rica and neighboring Central American countries.

## Data Availability

The data analyzed in this study were obtained from the Costa Rican Institute on Alcoholism and Drug Addiction (IAFA) and are not publicly available; access requires formal authorization from IAFA. Requests to access these datasets should be directed to https://iafa.go.cr/sobre-iafa/donde-estamos/#contacto.
